# Ferroptosis: A Critical Moderator in the Life Cycle of Immune Cells

**DOI:** 10.3389/fimmu.2022.877634

**Published:** 2022-05-10

**Authors:** Ping Wang, Yuan-Qiang Lu

**Affiliations:** ^1^ Department of Emergency Medicine, School of Medicine, The First Affiliated Hospital, Zhejiang University, Hangzhou, China; ^2^ Key Laboratory for Diagnosis and Treatment of Aging and Physic-chemical Injury Diseases of Zhejiang Province, Hangzhou, China

**Keywords:** ferroptosis, immune cell, iron metabolism, tumors, GPX4

## Abstract

Ferroptosis is a form of programmed cell death that was only recognized in 2012. Until recently, numerous researchers have turned their attention to the mechanism and function of ferroptosis. A large number of studies have shown potential links between cell ferroptosis and infection, inflammation, and tumor. At the same time, immune cells are vital players in these above-mentioned processes. To date, there is no comprehensive literature review to summarize the relationship between ferroptosis and immune cells. Therefore, it is of great significance to explore the functional relationship between the two. This review will attempt to explain the link between ferroptosis and various immune cells, as well as determine the role ferroptosis plays in infection, inflammation, and malignancies. From this, we may find the potential therapeutic targets of these diseases.

## Introduction

Over the last few decades, research has found a range of cell death mechanisms that are strictly regulated to maintain body healthy. Cell death is not only essential for normal development and homeostasis of the body, but also for the development and treatment of diseases associated with the immune system. Cell death and immunity maintain homeostasis through complex molecular and cellular interactions. Immune cells can prevent inflammation and autoimmune diseases by eliminating debris from dead cells; however, dead cells produce intracellular chemicals that provoke an immunological response.

Ferroptosis is defined as iron-dependent regulated necrosis-like cell death caused by membrane damage, which is mediated by a large amount of lipid peroxidation. Ferroptosis is different from other types of regulated cell death in morphology, biochemistry, and genetics. At the ultrastructural level, ferroptosis cells are abnormal in the mitochondria, such as condensation or swelling, increased membrane density, decreased or absent cristae, and rupture of the outer membrane (1, 2). At the same time, the size of the nucleus in ferroptotic cells did not change, but the integrity was destroyed, which caused the release of high mobility group protein B1 (HMGB1) (3).

In the fight against human diseases, especially inflammatory diseases, immune cells such as neutrophils, T lymphocytes, B lymphocytes, macrophages, natural killer (NK) cells, and dendritic cells (DC) all play important roles. The functions of different immune cells, or different stages of the same immune cell, are regulated by specific forms of cell death. Some studies have shown that ferroptosis is critical in regulating immune cell function. On the one hand, the ferroptosis of immune cells affect their number and function, thereby ferroptosis impairs the immune response. On the other hand, the ferroptosis of non-immune cells will lead to the release of damage-associated molecular pattern (DAMP) and initiate immune cell responses (4).

In this review, we will summarize how ferroptosis affects different types of immune cells in the cases of tumor, inflammation, and infection. This may provide directions for targeted therapeutic interventions.

## Iron Metabolism and Ferroptosis

As a basic inorganic nutrient for humans, iron participates in many biological processes. Both iron overload and iron deficiency can cause diseases, such as hereditary haemochromatosis, anemia, etc (5). The ferrireductase (DMT1) on the apical surface of duodenal and upper jejunum cells that converts Fe (III) to Fe (II). Some iron is weakly bound to ferritin, which comprises FTL (ferritin light chain) and FTH1 (ferritin heavy chain 1). Some iron forms a pool of iron termed the cytoplasmic labile iron pool (cLIP) (6), and some crosses the basolateral membrane through ferroportin (FPN) into the plasma to combine with transferrin (TF). Import, storage, and export of iron are tightly regulated and the regulation of iron homeostasis provides an integrated network of ferroptosis. Fe (III) can be recycled to Fe (II) *via* the Haber-Weiss reaction by oxidation with a peroxyl radical to oxygen. Excessive free reactive Fe (II) can accelerate the generation of deleterious ROS through the Fenton reaction (7). Then ROS interacts with PUFAs of lipid membranes and induces lipid peroxidation, which is indispensable for the ferroptosis (8).

## The Role of Autophagy In Ferroptosis

Autophagy is an evolutionary and conserved important process for the turnover of intracellular substances in eukaryotes (9). Moreover, autophagy has been reported to be necessary to control the dynamics of cell lipids and has a role in both the formation and degradation of lipid droplets (10, 11). In order to inhibit the oxidation of polyunsaturated fatty acids, free fatty acids are stored in lipid droplets. When lipid autophagy occurs, the lipid droplets are degraded, and the free fatty acids are released into cells which can promote the occurrence of ferroptosis (12). From the above, it is not difficult to find that autophagy is related to ferroptosis.

In addition, ferritinophagy is also related to ferroptosis. The term ferritinophagy is used to describe the degradation of transferrin and ferritin by the autophagy machinery, which releases Fe (II) into the labile iron pool and triggers labile iron overload, lipid peroxidation, membrane damage, and ferroptosis (13). Nuclear receptor coactivator 4 (NCOA4) is a selective cargo receptor which has been demonstrated it mediates the ferritinophagy (14). Genetic inhibition of NCOA4 inhibited ferritin degradation and suppressed ferroptosis. In contrast, overexpression of NCOA4 increased ferritin degradation and promoted ferroptosis (15). Hou et al. also found that the levels of Fe (II) and MDA were decreased in NCOA4-knockdown cells, whereas the levels of Fe (II) and MDA were increased in NCOA4-overexpressing cells (13).

## Major Pathways in Ferroptosis Regulation

**Figure 1:**
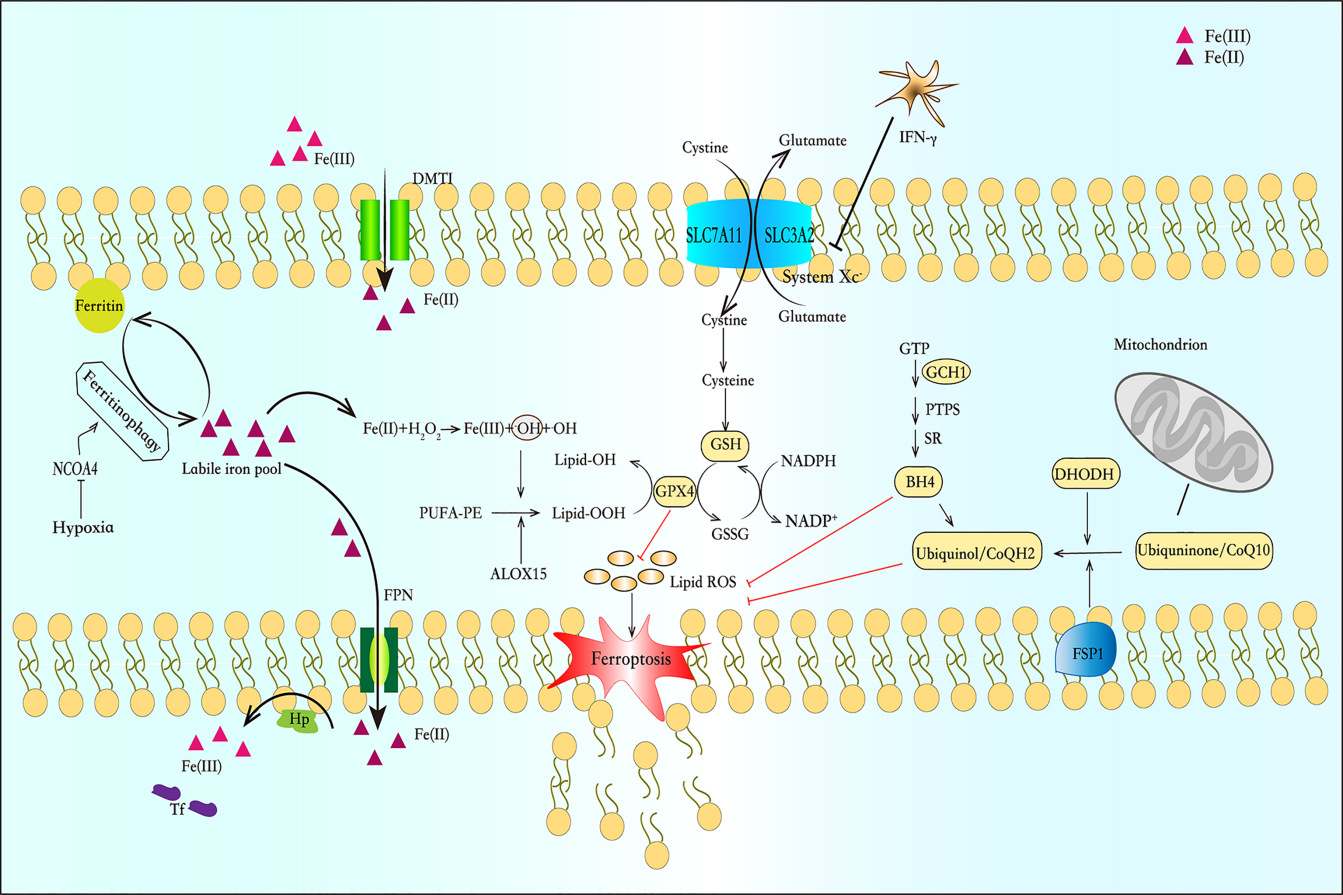
Iron metabolism and the pathway of ferroptosis. Ferrireductase (DMT1) converts Fe (III) to Fe (II). Some of Fe (II) is bound to ferritin and forms cytosolic labile iron pool (cLIP), and some crosses the basolateral membrane through ferroportin (FPN) into the plasma to combine with transferrin (TF). Fe (II) can initiate liposome peroxidation through the Fenton reaction. The system $x_{\text{c}}^{-}$ is a counter-transport that can ingest cystine and excrete glutamic acid to synthesize glutathione (GSH). Glutathione peroxidase 4 (GPX4) is a selenium enzyme that catalyzes GSH into oxidized glutathione (GSSG) and reduces toxic peroxides to non-toxic hydroxyl compounds. And the FSP1-CoQ-NADPH pathway and DHODH can inhibit lipid peroxidation and protect cells from ferroptosis by generating ubiquinol. In the GCH-BH4 pathway, GCH1 resists ferroptosis through its metabolite BH4. BH4 acts as a free-radical-trapping antioxidant or participates in ubiquinone synthesis to inhibit lipid peroxidation.

As mentioned above, ferroptosis is a type of cell death that depends on iron-mediated oxidative stress and lipid peroxidation-mediated cytotoxicity, and its main characteristics are iron accumulation and lipid peroxidation. Biochemically, ferroptosis is characterized by depletion of glutathione (GSH) and decreased expression of glutathione peroxidase 4 (GPX4). GSH-depletion caused by the inhibition of system $x_{\text{c}}^{-}$ is closely related to ferroptosis. The system $x_{\text{c}}^{-}$ , a counter-transport of cystine, involves anti-oxidative action by maintaining GSH level. GSH is one of the most important small-molecule antioxidants in cells. Several lines of evidence suggest that system $x_{\text{c}}^{-}$ might modulate the innate immune response and adaptive immune response. With excessive production of pathogen-toxic ROS, macrophages and granulocytes will up-regulate system $x_{\text{c}}^{-}$ expression. Secondly, it indirectly fosters GSH synthesis to lymphocytes and thereby regulates their proliferation and activation (16). With this feature, we can design ferroptosis potent inducers and inhibitors. Of course, a growing body of studies has suggested that the mechanisms of inducing and inhibiting ferroptosis are not limited to the GSH-GPX4 axis (**Figure 1**).

The FSP1-CoQ-NADPH pathway as a novel route for the suppression of phospholipid peroxidation and ferroptosis (17). FSP1 blocks lipid peroxidation and ferroptosis in a pathway independent of the proteins GPX4 and glutathione. FSP1 targets CoQ10 in the cell membrane to produce a reduced form of the molecule termed ubiquinol, which protects against lipid peroxidation that leads to ferroptosis. Whereas FSP1 catalyses the regeneration of CoQ10 using NAD(P)H (18). What’ s more, Dai et al. uncovered a mechanism independent of ubiquinol responsible for FSP1-mediated ferroptosis resistance, which modulates lipid peroxidation through an ESCRT-III–dependent membrane repair mechanism (19). In ferroptosis, ESCRT-III subunits (e.g., CHMP5 and CHMP6) accumulates in plasma membrane to antagonizes ferroptosis (20). Recently, Pedrera et al. demonstrated that ESCRT-III complexes are activated in a Ca2+-dependent manner to repair membrane damage, and ferroptosis can be delayed by osmoprotectants (21).

The GTP cyclohydrolase-1 (GCH1)-Tetrahydrobiopterin (BH4) pathway has been identified as another pathway that regulates ferroptosis. BH4 is an essential cofactor and plays multiple roles in the regulation of oxidative stress and inflammation (22). It can exert an anti-inflammatory effect by scavenging free radicals, but it is prone to auto-oxidation in the presence of molecular oxygen and therefore contributing to inflammatory processes (23) GCH1 is the first rate-limiting enzyme for BH4 biosynthesis. Studies have shown that GCH1 and BH4 prevent cells from lipid peroxidation damage during ferroptosis induction (24).Rrecent study revealed that the decrease of GCH1 and BH4 levels can promotes erastin-induced ferroptosis in colorectal cancer (CRC) cells, and this may be related to the reduction of NRF2, which needs to be further provn (25). This study provides a new therapeutic target for our use of ferroptosis inducers in the treatment of CRC. In addition, available evidence shows that GCH1 expression and BH4 synthesis are stimulated by immunological factors, notably pro-inflammatory cytokines, such as IL-1β, IFNγ, and TNFα, in response to pathogen invasion (26, 27). However, these results are mainly related to astrocytes, and there is still a lack of research on the relationship between GCH1-BH4 and immune cells.

DHODH, within the mitochondria, is an enzyme that generates ubiquinol by catalyzing the conversion of the molecule dihydroorotate (DHO) to orotate (OA). Mao et al. determined that DHODH coordinates with GPX4 to inhibit ferroptosis in the inner mitochondrial membrane. And DHODH deletion promotes ferroptosis, especially cancer cells that have low levels of expression of GPX4 (28).

**Figure 2:**
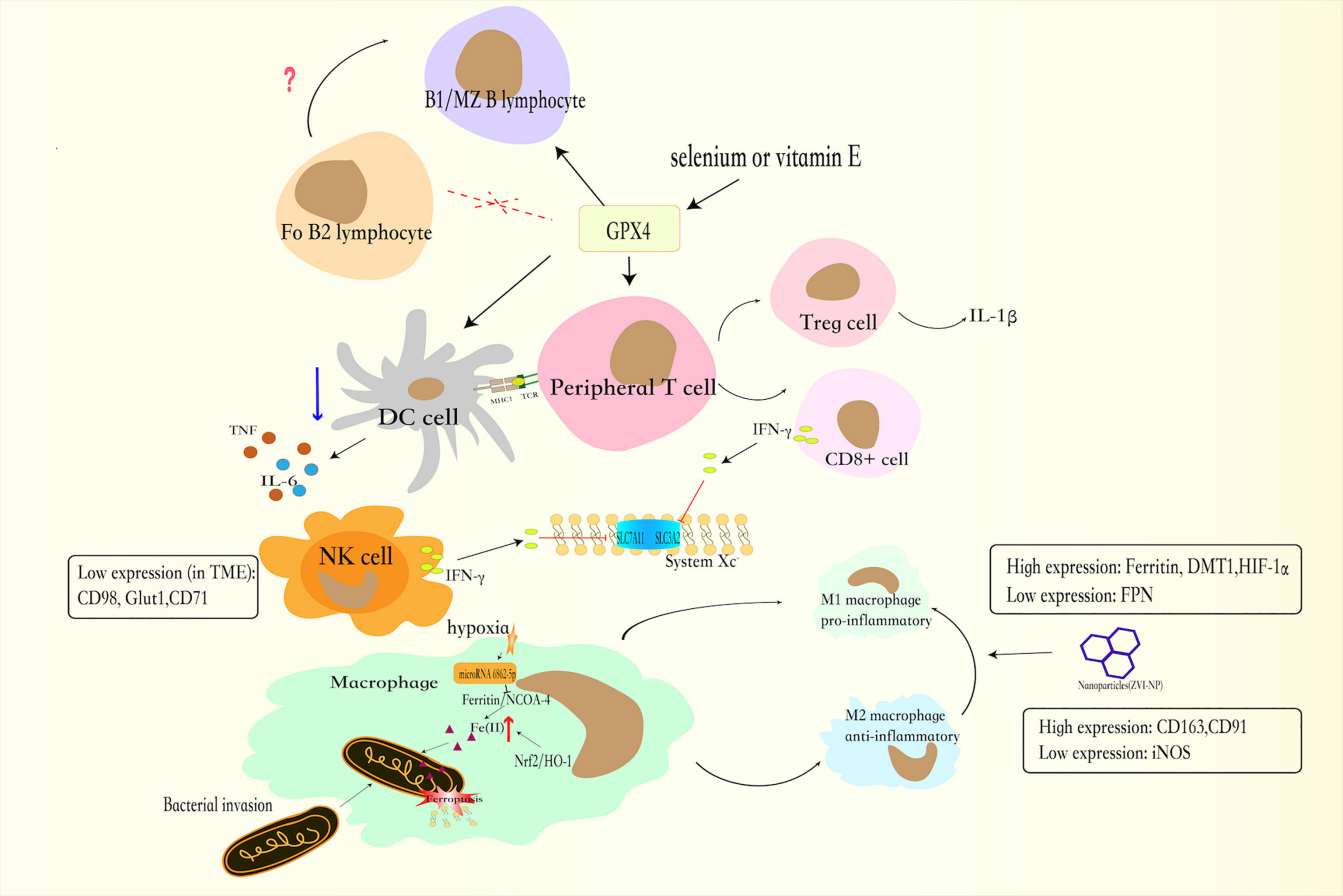
The GSH-GPX4 axis has differential effects in the life cycle of immune cells. The GPX4 is critical to the functions of peripheral T lymphocytes. CD8+ T lymphocytes secrete less IFNγ and Treg lymphocytes increases the production of IL-1β, which facilitates T helper 17 (TH17) responses. Together with CD8+ T lymphocytes, NK cells can produce IFNγ, which enhance the consumption of GSH, and increased the sensitivity of cell to ferroptosis. And NK cells, exposed to TME, have low expression of CD98, Glut1, and CD71 transferrin receptors. In the absence of GPX4, B1 and MZ B lymphocytes will cause ferroptosis. By contrast, Fo B2 lymphocytes do not require GPX4 for its maintenance. For DCs, GXP4 inhibitor blocks DCs maturation, weakens the ability of DCs to secrete TNF and IL6, and reduces MHC I expression.

Among all pathways which are mentioned above, GSH-GPX4 axis is the earliest discovered and best-researched pathway in ferroptosis, so our review mainly reveals the relationship between ferroptosis and immune cells from GSH-GPX4 axis (**Figure 2**). Perhaps this can provide inspiration for our further study of the relationship between ferroptosis and immune cells through other pathways.

## Immune Cells and Ferroptosis

### Ferroptosis Is Related to the Function of Neutrophils

Neutrophils are a group of complex immune cells with special functions (29). In the past, neutrophils were thought to be completely pro-inflammatory, but now existing evidences indicate that certain types of neutrophils exhibit anti-inflammatory or healing functions (30). Neutrophils are considered to combat invading microorganisms through the following three strategies: 1) phagocytosis, which involves the engulfment and subsequent elimination of microbes in specialized phagolysosome compartments; 2) degranulation, which is secreted antibacterial agents after infection; 3) the formation of neutrophil extracellular traps (NETs) (31). NETs are fibrous network structures released by neutrophils under the action of stimulating factors, consisting of decondensed chromatin and various antimicrobial proteins, such as elastase, cathepsin G, myeloperoxidase (MPO) and so on (32). After neutrophil death, NETs are still capable of enhancing bacterial killing. Sulfasalazine is a ferroptosis inducer that can perform its function by inhibiting cystine/glutamate antiporter SLC7A11 and activating arachidonate lipoxygenase (ALOX) (33). Sulfasalazine also accelerates NETosis by inducing the production of ether-linked oxidized phospholipids (a type of enzymatically oxidized phospholipids) (33). What’s more, it is well known that enzymatic oxidation of phospholipids is critical in regulating cell death and immunity (34). This hints a potential relationship between sulfasalazine-induced NETosis and sulfasalazine-induced ferroptosis. Fortunately, recent studies have shown that ether-linked glycerolipids promote the susceptibility to ferroptosis, which not only verifies the previous conjecture but also suggests that ether lipid may be the most important key link in the relationship between sulfasalazine-induced neutrophil NETosis and ferroptosis (35).

Ferroptosis is not only related to the formation of NETs, but to the anti-tumor, pro-inflammatory, and neutrophil-related autoimmune diseases. Studies found that in glioblastoma (GBM), neutrophils transfer particles containing MPO to tumor cells, which induce accumulation of lipid peroxides and iron in tumor cells, and trigger ferroptosis of tumor cells (36). The inflammatory response after heart transplantation is also related to ferroptosis and neutrophils, because the ferroptosis of graft endothelial cells promotes adhesion of neutrophils to coronary vascular endothelial cells through the TLR4/Trif/I type interferon (IFN) signaling pathway (37). Besides the connection between neutrophil ferroptosis and autoimmune development has not been thoroughly explored. But the findings in systemic lupus erythematosus (SLE) patients give us some enlightenment. The SLE patients often show a decrease in the number of neutrophils. Observing SLE patients’ neutrophil deaths, a small part can be attributed to NETosis, and a large part show ferroptosis (38). The theory that autoantibody and IF-α inhibit the expression of GPX4 resulting in ferroptosis of neutrophils may explain this phenomenon (39). This suggests that inhibiting neutrophil ferroptosis may improve disease severity of patients with SLE.

### The Role of Macrophages in Iron Metabolism and Ferroptosis

Macrophages play an important physiological role in iron metabolism and circulation. Splenic red pulp macrophages (RPMs) and liver macrophages (Kupffer cells) are used exclusively for iron homeostasis (40). Among them, splenic RPMs rely on the activity of heme-responsive Spi-C transcription factor to control red blood cell (RBC) circulation and iron homeostasis (41). On average, each macrophage clears one RBC per day and recovers iron from it. When the number of RBCs that need to be cleared increases appropriately, macrophages can inhibit the cytotoxic effect of free heme by increasing the expression of heme oxygenase-1 (HO-1). Meanwhile, when the number of RBCs that need to be exceeded a certain level, the efficiency and quantity of HO-1 expression may not be enough to prevent cell death (42). In mouse models that mimicked RBC transfusion and clearance in a clinical setting, the RBC phagocytosis of splenic RPMs increased after rapid transfusion of large amounts of RBCs, but the increase in HO-1 was still insufficient to prevent ferroptosis of splenic RPMs triggered by massive RBC phagocytosis (43).

It has been reported that the inhibition of iron output and/or the increase of uptake leads to the iron entering the cytosolic labile iron pool (cLIP) and induces ferroptosis (44). Fortunately, macrophages have a certain resistance to iron overload. Studies have shown that during hypoxia, microRNA 6862-5p degrades the mRNA of NCOA4, resulting in a decrease in the expression of NCOA4, increasing the levels of mitochondrial ferritin (FTMT) and FTH which protect macrophages from ferroptosis (45).

The recent research indicates that ferroptotic stress is well controlled in macrophages upon bacterial infection. Macrophages can increase intracellular Fe (II) by activating NRF2/HO-1 and ferritin/NCOA-4, and by reducing FPN-mediated efflux. At the same time, macrophages protect cells from ferroptotic damage by instigating GPX4 expression. On the other hand, the increasing intracellular Fe (II) can be transported into bacterial-containing vesicles by FPN, inducing bacterial ferroptosis. At the cellular level, adding ferroptosis inducers increased lipid ROS levels and helped in macrophage defense against intracellular bacteria (46). Therefore, targeting the regulation of ferroptosis stress may be a potential strategy for the treatment of intracellular pathogen infections.

Different polarized macrophages have different performances in iron metabolism and ferroptosis. For example, in glial cells (brain macrophages), compared with M2 microglias, M1 cells are more resistant to ferroptosis resulting from the loss of arachidonic acid 15-lipoxygenase (ALOX15) activity (47). Under different stimulations, macrophages can be polarized into different subtypes, including broad categories: pro-inflammatory (M1) macrophages and alternative anti-inflammatory (M2) macrophages (48). Iron chelation in M1 macrophages is thought to be crucial to host defense. M1 phenotype expresses high levels of ferritin, divalent metal transporter 1 (DMT1), and hypoxia-inducible factor 1α (HIF-1α), but expresses low levels of transferrin receptor and FPN to maximize iron absorption and storage, and down-regulate iron output (49). It has been shown to have higher resistance to ferroptosis induced by GPX4 deletion than M2 (50). In terms of mechanism, M1 phenotype produces NO free radicals (NO•), which causes the inhibition of secondary ALOX15 activity, while iNOS expression and NO• production in M2 phenotype are inhibited (51). In addition to this, the expression of iron metabolism-related proteins in M2 phenotypes is different. M2 phenotype shows increased phagocytic activity and high expression of scavenging receptors such as CD163 and CD91 to release iron, which is conducive to affect tissue regeneration, healing (52), and the growth of tumor cells (53). It is known that tumor-associated macrophages (TAMs) in the tumor microenvironment (TME) are actually M2-like polarized macrophages, which can suppress anti-tumor immunity (54). Therefore, the elimination of M2 phenotype or repolarization of M2 phenotype to M1 in TAM has gained popularity in the field of cancer immunotherapy. Hsieh et al. found that Zero-valent-iron (ZVI) nanoparticle (NP) can functions like a ferroptosis inducer. It can be rapidly converted to Fe (III) in the lysosomes of cancer cells and generate massive ROS, which triggers ferroptosis. And under the cancer co-culture system, ZVI-NP also efficiently repolarizes macrophages from M2 phenotype to M1 phenotype (55). Many nanoparticles have also been demonstrated to utilize the above two mechanisms for tumor immunotherapy (56, 57), but evidence that ferroptosis inducers can directly modulate macrophage repolarization in the tumor microenvironment is lacking. At the same time, in the non-tumor environment, the relationship between ferroptosis inducers and macrophage repolarization is still blank.

### Ferroptosis in Different Subtypes of T Lymphocytes

T lymphocytes are derived from bone marrow progenitor cells, which migrate to the thymus for maturation, selection, and subsequent export to the periphery. Peripheral T lymphocytes include different subpopulations: toxic T cells, helper T cells, regulatory T cells, and memory T cells (58). GPX4 degrades small molecular peroxides and certain lipid peroxides, thereby protecting the structure and function of cell membranes from the interference and damage of peroxidation (59). A previous study of ferroptosis in the T lymphocytes found that GPX4-deficient T lymphocytes developed normally in the thymus, but the peripheral CD8^+^ T and CD4^+^ T functions were impaired (60). Subsequent studies have found that cystine and cysteine transporters are not expressed or are low-expressed in naïve T lymphocytes. However, these transporter expressions are up-regulated when T lymphocytes are activated (61). This is because the proliferation of activated T lymphocytes requires higher bioenergy, which is mainly provided by glucose and glutamine. These high metabolic activities produce more ROS, which in turn require endogenous antioxidants to stabilize the cells, especially GSH. Therefore, although GSH-deficient T lymphocytes will be activated normally at first, they cannot reprogram their metabolism to meet their increasing energy requirements, resulting in T lymphocytes ferroptosis and causing immune dysfunction (62). As mentioned earlier, GPX4 is a selenium enzyme, and further studies have found that selenium supplementation can increase the level of GPX4 and enable follicular helper T (TFH) lymphocytes to produce high-affinity and long-lived humoral immunity (63). This gives us new ideas for the treatment of T cell-related diseases. For example, whether we can protect and enhance T lymphocytes’ immunity by ingesting selenium or vitamin E.

Not only the activation and proliferation of T lymphocytes are related to ferroptosis, but the anti-tumor ability of T lymphocytes is also closely related to ferroptosis. On the one hand, CD8^+^ T lymphocytes can secrete IFNγ to downregulate the expression of SLC3A2 and SLC7A11, and as a consequence, promote lipid peroxidation and ferroptosis of tumor cell (64). On the other hand, in TME, a large amount of lipid peroxidation can be detected in CD8^+^ T lymphocytes. Thus, the ferroptosis of CD8^+^ T lymphocytes is increased, which results in a decrease in the secretion of killer factors and a weakened anti-tumor function. This may be related to increased expression of CD36 in CD8^+^ T lymphocytes (65).

The paradoxical effects of ferroptosis on tumor cells and T cells have created obstacles for us to adopt anti-tumor therapy by inducing ferroptosis of tumor cells. Fortunately, several studies have revealed that the ways of inducing ferroptosis of tumor cells and CD8^+^ T lymphocytes are different. In addition, the sensitivities of these two cell types to ferroptosis inducers are also different. The sensitivity of tumor cells is higher than that of activated CD8^+^ T lymphocytes, and some ferroptosis inducers of tumor cells do not damage CD8^+^ T lymphocytes, such as erastin and RSL3 (64). Therefore, more research is needed to discover the significance of ferroptosis in tumor treatment. Targeting tumor ferroptosis-related metabolism through the intervention of CD36 may be an effective strategy for improving the anti-tumor efficacy of CD8^+^ T lymphocyte immunotherapy, which provides a new option for clinical treatment.

As a special T lymphocyte subset, Treg lymphocytes have immunosuppressive effects. Treg lymphocytes secrete more thioredoxin-1 (an antioxidant) than effector T lymphocytes, as a result, Treg lymphocytes are more resistant to ROS-induced ferroptosis (66). GPX4-deficient Treg lymphocytes can cause Treg lymphocytes ferroptosis upon T cell receptor (TCR)/CD28 co-stimulation. Moreover, it also increases the production of interleukin-1β (IL-1β), which facilitates T helper 17 (TH17) responses. Furthermore, GPX4-deficient Treg lymphocytes can decrease the number of Treg lymphocytes in TME and the enhance the anti-tumor immune response, thereby repressing tumor growth (67). Therefore, the induction of excessive accumulation of lipid peroxidation and ferroptosis in Treg lymphocytes may promote the development of therapeutic strategies in cancer treatment.

### B Lymphocytes Respond Differently to Ferroptosis, Which May Be Related to Autophagy

Three classes of B cells are produced during ontogeny: B1 lineage, B2 lineage follicular (Fo), and marginal zone (MZ) B cells. The B1 lineage is divided into B1a and B1b subgroups, which are considered the main source of natural circulating immunoglobulin. Studies have shown that the requirements of GPX4 pathway in T and B lymphocytes are different. We have previously described the importance of GPX4 upon T lymphocytes. Here we mainly describe the importance of GPX4 for B lymphocytes. Different subpopulations of B lymphocytes give various feedback on GPX4 deletion due to the difference in lipid metabolism between Fo B2 lymphocytes and B1/MZ B lymphocyte. The ferroptosis of B1 and MZ B lymphocytes is primed by the toxic effect of lipid peroxide accumulation in the absence of GPX4 (68). By contrast, Fo B2 lymphocytes do not require GPX4 for its development, homeostatic maintenance, germinal center reactions, and antibody responses (69). Furthermore, after an acquisition of B1 cell-typical self-reactive B lymphocyte receptor through proliferation, B2 lymphocytes can differentiate into functional B1 lymphocytes (70). This interesting discovery provides us with new strategy for immunotherapy. To test whether the conversion of B2 to B1 lymphocytes will also change their metabolic characteristics and affect their demand for GPX4 needs further study.

Autophagy may regulate the function of B lymphocytes through ferroptosis, because B1 B lymphocytes are more biologically active than B2 B lymphocytes, and compared with Fo B2 lymphocytes, the survival and self-renewal of B1a B lymphocytes are more dependent on the uptake of source fatty acids and mobilization of autophagy (71).

Ferroptosis is also of great significance in the treatment strategy of B lymphocytes immune-related diseases. In mouse lymphoma models, erastin can induce ferroptosis by inhibiting the system $x_{\text{c}}^{-}$ and slowing down the tumor growth (72). In germinal center B-cell diffuse large B-cell lymphoma (DLBCL), dimethyl fumarate (DMF) reduces GSH and GPX4 levels and increases the expression of 5-lipoxygenase, which induces ferroptosis. In activated B-cell DLBCL, DMF induces the succination of kinases IKK2 and JAK1, thereby inhibiting NF-κB and JAK/STAT survival signaling (73). Analysis of protein-protein interaction network also shows that ferroptosis may be closely related to B lymphocytes receptor signaling and the production of IgA in the immune-deficient intestinal tract (74). To date, there is a lack of evidence showing an association between ferroptosis and tumor-infiltrating B lymphocytes.

### NK Cells Release IFNγ to Induce Ferroptosis

Unlike T and B cells, NK cells can non-specifically kill tumor cells and virus-infected cells without pre-sensitization. Together with CD8^+^ T lymphocytes, NK cells are effective producers of IFNγ (75). IFNγ affects the sensitivity of cells to ferroptosis. A study investigated the effect of IFNγ on hepatocellular carcinoma (HCC) found that IFNγ enhanced the consumption of GSH, increased lipid peroxidation, and increased the sensitivity of HCC to ferroptosis. At the same time, IFNγ induces ferroptosis in HCC by activating JAK/STAT signal transduction and inhibiting system $x_{\text{c}}^{-}$ (76). But in TME, NK cells dysfunction occurs. A recent study showed that this might be related to ferroptosis. The study found that the expressions of CD98, Glut1, and CD71 transferrin receptors in NK cells decreased when NK cells were exposed to TME. The expressions of proteins related to ferroptosis, lipid peroxidation, and oxidative damage increased (77). Therefore, we can further explore the effects of ferroptosis inducers and inhibitors on the survival and function of tumor-related NK cells in subsequent experiments.

### Ferroptosis Leads to the Dysmaturity and Dysfunction of DC Cells

DCs are at the center of initiating, regulating, and maintaining the immune response. Immature DCs have strong antigen phagocytic ability and differentiate into mature DCs when they are stimulated by antigen. After that, DCs will contact T cells and stimulate an immune response. Thus DCs is regarded as the most effective antigen-presenting cell (APC) (78). The nuclear hormone receptor peroxisome proliferator-activated receptor γ (PPARγ) enables cells to shape immunity according to their lipid environment (79). Studies have found that RSL3 (GXP4 inhibitor) can be responsible for inducing ferroptosis through PPARG, which blocks DCs maturation, weakens the ability of DCs to secrete TNF and IL6, and reduces MHC class I expression in response to lipopolysaccharide signals. In addition, after ferroptosis happens to DCs, they cannot induce CD8^+^ T cells to produce IFNγ (80). It is well known that the antigen presentation ability and anti-tumor ability of DCs decrease in TME, it may result from the occurrence of ferroptosis induced by lipid accumulation in DCs (81).

In addition, the pathogenesis of sepsis is also related to the ferroptosis of DCs. A functional exhaustion of DCs will promote septic complications during sepsis. Fortunately, the immune system has a protection mechanism. In the late stage of sepsis, macrophages will release high mobility group protein B1 (HMGB1), which can stimulate the expression of Sesn2 in DCs (82). Sesn2 inhibits the ferroptosis of DCs in sepsis by down-regulating the ATF4-CHOP-CHAC1 signaling pathway and exerts an antioxidant effect (83). This discovery inspires us that exploring the relationship between sepsis and ferroptosis may help us overcome sepsis, a disease that seriously threatens human health.

## Conclusion

Ferroptosis is closely related to immune cells. The regulatory pathways and mechanisms of ferroptosis in different immune cells are also various, and they are not limited to the glutathione-GPX4 axis. It implies that the relationship between ferroptosis and immune cells need to be examined from other pathways. And this review is of great significance to further explore the relationships between ferroptosis and tumor, infection, and inflammation. However, we have to admit that there are still many unknown factors in the immune effect of ferroptosis. For example, we know that the uptake of apoptotic bodies can lead to immunosuppression (84). The immune function of the bubbles released during ferroptosis is still unclear and needs further investigation. Furthermore, how different immune cell subgroups perceive ferroptosis and then amplify or weaken the subsequent response is worthy of our continuing exploration. In the end, the clinical application value of ferroptosis in immune cells need us to further explore.

## Author Contributions

PW conceived and completed the manuscript. Y-QL supervised and revised the manuscript. All authors contributed to the article and approved the submitted version.

## Funding

This study was sponsored by the Foundation of Key Discipline Construction of Zhejiang Province for Traditional Chinese Medicine (No. 2017-XK-A36) and the Opening Foundation of State Key Laboratory for the Diagnosis and Treatment of Infectious Diseases (No. 2018KF02)

## Conflict of Interest

The authors declare that the research was conducted in the absence of any commercial or financial relationships that could be construed as a potential conflict of interest.

## Publisher’s Note

All claims expressed in this article are solely those of the authors and do not necessarily represent those of their affiliated organizations, or those of the publisher, the editors and the reviewers. Any product that may be evaluated in this article, or claim that may be made by its manufacturer, is not guaranteed or endorsed by the publisher.
